# A novel approach to solve nonlinear Fredholm integral equations of the second kind

**DOI:** 10.1186/s40064-016-1810-8

**Published:** 2016-02-24

**Authors:** Hu Li, Jin Huang

**Affiliations:** School of Mathematical sciences, University of Electronic Science and Technology of China, Chengdu, 611731 Sichuan People’s Republic of China

**Keywords:** A novel approach, Nonlinear Fredholm integral equations, Integral mean value theorem, Newton iteration

## Abstract

In this paper, we present a novel approach to solve nonlinear Fredholm integral equations of the second kind. This algorithm is constructed by the integral mean value theorem and Newton iteration. Convergence and error analysis of the numerical solutions are given. Moreover, Numerical examples show the algorithm is very effective and simple.

## Background

Integral equations have several applications in Physics and Engineering. However, these occur nonlinearly. In particular, nonlinear integral equations arise in fluid mechanics, biological models, solid state physics, kinetics in chemistry etc. In most cases, it is difficult to solve them, especially analytically.

In the past several years, the nonlinear integral equations have been solved numerically by several workers, utilizing various approximate methods (see Atkinson and Potra 1988; Atkinson and Flores 1993; Babolian and Shahsavaran 2009; Lepik and Tamme 2007; Saberi-Nadjafi and Heidari 2010; Aziz and Islam 2013; Maleknejad and Nedaiasl 2011).

In the present work, we have developed a novel approach to solve nonlinear Fredholm integral equations of the second. This algorithm is obtained by integral mean value theorem and Newton iteration. We consider the nonlinear Fredholm integral equations, given as follows:

1 $$u(x)=f(x)+\int _a^bK(x,y)g(u(y))dy, \quad x\in [a,b],$$

where *f*(*x*) is a known continuous function defined on [*a*, *b*] and *g*(*u*(*y*)) is a nonlinear function defined on [*a*, *b*]. The nonlinear integral operator *k* is defined as follows:

2 $$(kg(u))(x)=\int _a^bK(x,y)g(u(y))dy,\quad x\in [a,b],$$

and *k* is compact on *C*[*a*, *b*] into *C*[*a*, *b*] with continuous kernel *K*(*x*, *y*). Then (1) is equivalent to the operator form as follows:

3 $$u-kg(u)=f.$$

This paper is organized as follows: In section “A novel numerical method”, based on the idea of the integral mean value theorem, a novel numerical method is given. In section “Convergence and error analysis”, we address the convergence and error analysis of the numerical solutions. In section “Description of Newton iteration and a novel algorithm”, Newton iteration is introduced and a novel algorithm is given. In section “Numerical results”, numerical examples are carried out.

## A novel numerical method

In order to obtain a novel numerical method, we firstly introduce the integral mean value theorem, is given as follows:

### **Theorem 1**

*If s*(*x*) *is continuous on the closed interval* [*a*, *b*]*, there is a number*$c\in [a,b]$*so that*

4 $$M(s)=\int _a^bs(x)dx=(b-a)s(c).$$

Let $h=(b-a)/n, n\in N$ be the mesh with $x_k=a+kh, k=0,\ldots , n$. By (4), we can construct a sequence of quadrature formula as

5 $$M(s,c_k)=\sum _{k=0}^{n-1}\int _{x_k}^{x_{k+1}}s(x)dx=h\sum _{k=0}^{n-1}s(x_k+hc_k),\quad 0<c_k<1,$$

where $c_k,(k=0,\ldots ,n-1)$ are constants.

We apply (5) to the integral operator *K* and get

6 $$(kg(u))(x)=h\sum _{k=0}^{n-1}K(x,x_k+hc_k(x))g(u(x_k+hc_k(x))),\quad x\in [a,b],$$

where the unknown function $c_k(x),(k=0,\ldots ,n-1)$ are dependent on the variable *x* and $0<c_k(x)<1$. Especially, Let $c_k(x)=c_k$ be constants. We can obtain Nyström approximation with a high accuracy, is given as follows:

7 $$(k_ng(u))(x)=h\sum _{k=0}^{n-1}K(x,x_k+hc_k)g(u(x_k+hc_k)),\quad x\in [a,b], \quad 0<c_k<1.$$

Thus we obtain the numerical approximate form of (3)

8 $$u_n-k_ng(u_n)=f.$$

Obviously, Eq. (8) is a nonlinear equations system. Once $u_n$ is get, we obtain $u(x), x\in [a,b]$ by (3).

## Convergence and error analysis

We give the convergence analysis of (8) and have a theorem as follows:

### **Theorem 2**

*If the function K(x,y) is continuous on*$[a,b]\times [a,b]$*and g(x) is continuous on [a, b], they satisfy the following Lipschitz conditions*

$$\begin{aligned}&\Vert K(x,y_1)-K(x,y_2)\Vert _{\infty }\le L_1\Vert y_1-y_2\Vert _{\infty },\\&\Vert g(x_1)-g(x_2)\Vert _{\infty }\le L_2\Vert x_1-x_2\Vert _{\infty },\\&\Vert u(x_1)-u(x_2)\Vert _{\infty }\le L_3\Vert x_1-x_2\Vert _{\infty }, \end{aligned}$$

*with the constants*$L_{1,2,3}>0$*, the sequence*$(k_ng(u))(x)$*of quadrature formula is convergent. That is, we have*

$$(k_ng(u))(x)\rightarrow (kg(u))(x)=\int _a^bK(x,y)g(u(y))dy,\quad n\rightarrow \infty .$$

### *Proof*

By (6) and (7), we easily get

$$\begin{aligned}&\Vert (k_ng(u))(x)-(kg(u))(x)\Vert _{\infty }\\&\quad \le h\sum _{k=0}^{n-1}\left\| K(x,x_k+hc_k)g(u(x_k+hc_k))-K(x,x_k+hc_k(x))g(u(x_k+hc_k(x)))\right\| _{\infty }\\&\quad =h\sum _{k=0}^{n-1}\Vert K(x,x_k+hc_k)g(u(x_k+hc_k))-K(x,x_k+hc_k)g(u(x_k+hc_k(x)))\\&\qquad +K(x,x_k+hc_k)g(u(x_k+hc_k(x)))-K(x,x_k+hc_k(x))g(u(x_k+hc_k(x)))\Vert _{\infty }\\&\quad \le h\sum _{k=0}^{n-1}\Vert K(x,x_k+hc_k)g(u(x_k+hc_k))-K(x,x_k+hc_k)g(u(x_k+hc_k(x)))\Vert _{\infty }\\&\qquad +h\sum _{k=0}^{n-1}\Vert K(x,x_k+hc_k)g(u(x_k+hc_k(x)))-K(x,x_k+hc_k(x))g(u(x_k+hc_k(x)))\Vert _{\infty }\\&\quad \le h^2\left[ L_2L_3\max _{a\le x,y\le b}|K(x,y)|+L_1\Vert g(u(x))\Vert _{\infty }\right] \sum _{k=0}^{n-1}\Vert c_k-c_k(x)\Vert _{\infty }\\&\quad \le \frac{(b-a)^2}{n}\left[ L_2L_3\max _{a\le x,y\le b}|K(x,y)|+L_1\Vert g(u(x))\Vert _{\infty }\right] , \end{aligned}$$

where $0<c_k<1$ and $0<c_k(x)<1$. We have $\Vert (k_ng(u))(x)-(kg(u))(x)\Vert _{\infty }\rightarrow 0, n\rightarrow \infty$, and the proof of the theorem is completed. $\square$

From Theorem 2, we can get a corollary as follows:

### **Corollary 1**

*Under the assumption of Theorem*2*, the error of the approximate solutions in* (8) *can be estimated, is given as follows:*

9 $$\Vert u_n(x)-u(x)\Vert _{\infty }\le \frac{(b-a)^2}{n}\left[ L_2L_3\max _{a\le x,y\le b}|K(x,y)|+L_1\Vert g(u(x))\Vert _{\infty }\right] .$$

## Description of Newton iteration and a novel algorithm

We shall give Newton iteration to solve nonlinear equations. For convenience, we denote

10 $$\Psi (z)=(\varphi _0(z),\ldots ,\varphi _{n-1}(z)),$$

where $$z=(z_0,\ldots ,z_{n-1})^T=u_n$$, and

11 $$\varphi _i(z)=z_i-h\sum _{j=0}^{n-1}K_{i,j}g(z_j)-f_i,\quad i=0,\ldots ,n-1$$

with $K_{i,j}=K(x_i+hc_i,x_j+hc_j)$. Then, (8) can be rewritten as

12 $$\Psi (z)=0.$$

The Jaccobi matrix of $\Psi (z)$ is

13 $$A(z)=\Psi ^{'}(z)=(\partial _j\varphi _i(z))_{n\times n}.$$

So New iteration is constructed

14 $$z^{l+1}=\omega (z^l), \omega (z)=z-(A(z))^{-1}\Psi (z),\quad l=0,1,2,\ldots$$

### **Lemma 1**

[Ostrowski see Ortege and Kheinboldt (1970)] *Suppose there is a fixed point*$z^{*}\in int(D)$*of the mapping:*$\omega : D\subset R^n\rightarrow R^n$*and the F-derivation of*$\omega$*at point*$z^{*}$*exists. If the spectral radius of*$\omega ^{'}(z^{*})$*satisfies*

15 $$\rho (\omega ^{'}(z^{*}))=\delta <1.$$

*Then, there is an open ball*$S=S(z^{*},\delta _{0})\subset D$*that for*$z^{0}\in S$*, the iterative sequence* (14) *is stable and convergent to*$z^{*}$.

### **Lemma 2**

[see Ortege and Kheinboldt (1970)] *Suppose*$A, C\in L(R^n), \Vert A^{-1}\Vert <\beta , \Vert A-C\Vert <\alpha , \alpha \beta <1,$*then C is invertible and*$\Vert C^{-1}\Vert <\beta /(1-\alpha \beta )$.

### **Theorem 3**

*Suppose*$\Psi : D\subset R^n\rightarrow R^n$*is F-derivative, and*$z^{*}$*satisfies equation*$\Psi (z)=0$. $A: S\subset D\rightarrow L(R^n)$*is continuous and invertible at*$z^{*}$, *where S is the neighborhood of*$z^{*}$*. Then, there is a close ball*$\bar{S}=\bar{S}(z^{*},\delta )\subset S$*that*$\Omega$ is *F-derivative at*$z^{*}$:

16 $$\omega ^{'}\left( z^{*}\right) =I-\left( A(z^{*})\right) ^{-1}\Psi ^{'}\left( z^{*}\right) .$$

### *Proof*

Let $\beta =\Vert (A(z^{*}))^{-1}\Vert >0$. Since $A(z^{*})$ is invertible, and *A*(*z*) is continuous at $z^{*}$, for $0<\varepsilon <(2\beta )^{-1}, \exists \delta >0,$ when $z\in \bar{S}(z^{*},\delta ),$ there is $\Vert A(z)-A(z^{*})\Vert <\varepsilon.$ According to Lemma 2, $(A(z))^{-1}$ exists and $\Vert (A(z))^{-1}\Vert \le \beta /(1-\varepsilon \beta )$ for any $z\in \bar{S}.$ So we construct the function

$$\omega (z)=z-(A(z))^{-1}\Psi (z), \quad z\in \bar{S}.$$

Since $\Psi (z)$ is derivative at $z^{*}, \exists \delta >0.$ When $z\in \bar{S}(z^{*},\delta ),$ we obtain an inequality by the definition of the *F-derivation*:

17 $$\left\| \Psi (z)-\Psi \left( z^{*}\right) -\Psi ^{'}\left( z^{*}\right) \left( z-z^{*}\right) \right\| \le \varepsilon \left\| z-z^{*}\right\| .$$

Consider the derivation of $\omega (z)$

$$\begin{aligned}&\left\| \omega (z)-\omega (z^{*})-\left[ I-(A(z^{*}))^{-1}\Psi ^{'}(z^{*})\right] (z-z^{*})\right\| \\&\quad =\left\| -(A(z))^{-1}\Psi (z)-(A(z^{*}))^{-1}\Psi ^{'}(z^{*})(z-z^{*})\right\| \\&\quad \le \left\| (A(z))^{-1}(A(z^{*})-A(z))(A(z^{*}))^{-1}\Psi ^{'}(z^{*})(z-z^{*})\right\| \\&\qquad +\left\| (A(z))^{-1}(\Psi (z)-\Psi (z^{*})-\Psi ^{'}(z^{*})(z-z^{*}))\right\| \\&\quad \le \left( 2\beta ^2\varepsilon \left\| \Psi ^{'}(z^{*})\right\| +2\beta \varepsilon \right) \le c\varepsilon \Vert z-z^{*}\Vert , \end{aligned}$$

where $c=2\beta (\beta \Vert \Psi ^{'}(z^{*})\Vert +1)$. According to the definition of the *F-derivation*, we obtain the the *F-derivation* of $\omega$ at $z^{*}$

$$\omega ^{'}(z^{*})=I-(A(z^{*}))^{-1}\Psi ^{'}(z^{*}).$$

Using the definition of the matrix *A* in (13), we have $\rho (\omega ^{'}(z^{*}))=0<1$. According to Lemma 1, the iterative sequence is stable and convergent to $z^{*}$.

In what follows, in order to give the numerical solutions with more stability, we provide a novel algorithm (see Zhong 2013).

- **Step 1** Take *n* and Let $x_k=a+hk,(k=0,\ldots ,n-1)$ with $h=(b-a)/n.$
- **Step 2** Let $c_k=c, (k=0,\ldots ,n-1)$ and randomly choose a series of $\sigma _i$ so that $0<c=\sigma _i<1, (i=0,1,\ldots ,m)$.
- **Step 3** Solve the nonlinear system by Newton iteration
  $$u_n^j-h\sum _{k=0}^{n-1}K(x_j+h\sigma _i,x_k+h\sigma _i)g\left( u_n^k\right) =f(x_j+h\sigma _i).$$
- **Step 4** Get the approximate solutions
  $$u_n(x,\sigma _i)=f(x)+h\sum _{k=0}^{n-1}K(x,x_k+h\sigma _i)g\left( u_n^k\right) .$$
- **Step 5** Let the mean value of $u_n(x,\sigma _i)$ be the last approximate solution
  $$u_n(x)=\sum _{i=0}^m\frac{u_n(x,\sigma _i)}{m+1}.$$

$$\square$$

## Numerical results

In this section, the theoretical results of the previous section are used for some numerical examples.

### *Example 1*

The following nonlinear integral equation is considered

$$u(x)=x\int _0^1y\sqrt{u(y)}dy+2-\frac{1}{3}(2\sqrt{2}-1)x-x^2,$$

with $0<x<1$ and the exact solution $u(x)=2-x^2.$

**Fig. 1:**
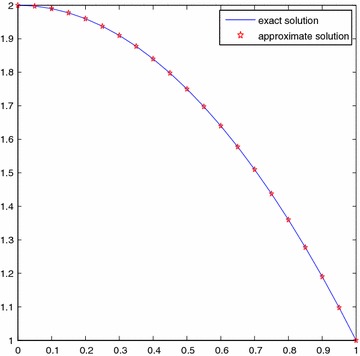
Comparison of solutions for Example 1

**Fig. 2:**
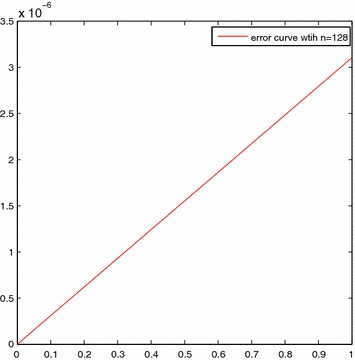
The error curve for Example 1

For the sake of simplicity, we choose $\sigma _i=i/10, (i=0,1,\ldots ,10)$. Table 1 shows the three kinds results by using the methods in Lepik and Tamme (2007), Aziz and Islam (2013), and the present method, respectively. Figure 1 shows the comparison of approximate and exact solutions with *n* = 128 and Fig. 2 presents the error curve on [0, 1] with *n* = 128.

**Table 1:** Absolute errors for Example 1

*x*	*n* = 8	*n* = 16	*n* = 32	*n* = 64	*n* = 128
0.2	1.63e−3	4.09e−4	9.94e−5	2.48e−6	6.21e−7
0.4	3.27e−3	8.18e−4	1.99e−4	4.97e−6	1.24e−6
0.6	4.90e−3	1.23e−3	2.98e−4	7.45e−6	1.86e−6
0.8	6.54e−3	1.64e−3	3.97e−4	9.94e−6	2.48e−6
Results in Aziz and Islam (2013)	1.0e−3	2.6e−4	6.6e−5	1.7e−5	4.2e−6
Results in Lepik and Tamme (2007)	2.7e−3	1.1e−3	3.7e−4	1.1e−4	3.1e−5

### *Example 2*

The following nonlinear integral equation is considered

$$u(x)=\frac{1}{5}\int _0^1\cos (\pi x)\sin (\pi y)[u(y)]^3dy+\sin (\pi x),$$

with $0<x<1$ and the exact solution $u(x)=\sin (\pi x)+\frac{1}{3}(20-\sqrt{391})\cos (\pi x)$.

**Fig. 3:**
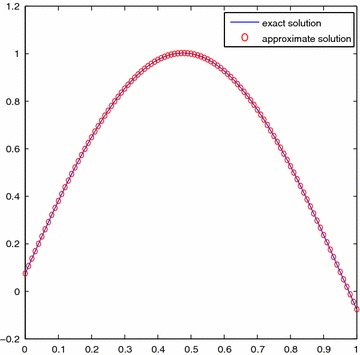
Comparison of solutions for Example 2

**Fig. 4:**
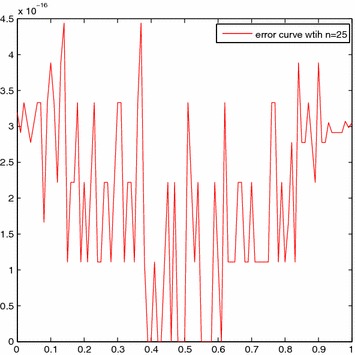
The error curve for Example 2

We take *n* = 25 along with h = 1/25 and get $x_k=k/25, (k=0,1,\ldots ,24)$. For the sake of simplicity, $\sigma _i$ is given as $i/10, (i=0,1,\ldots ,10)$. Table 2 shows the four kinds results by using Newton–Kantorovich-quadrature method in Saberi-Nadjafi and Heidari (2010), the SE-Sinc method in Maleknejad and Nedaiasl (2011), the DE-Sinc method in Maleknejad and Nedaiasl (2011), and the present method, respectively. Figure 3 shows the comparison of approximate and exact solutions with *n* = 25 and Fig. 4 presents the error curve on [0, 1] with *n* = 25.

**Table 2:** Absolute errors for Example 2

*x*	Results in Saberi-Nadjafi and Heidari (2010)	s-results in Maleknejad and Nedaiasl (2011)	d-results in Maleknejad and Nedaiasl (2011)	Present method
0	4.98e−2	4.15e−7	1.40e−10	3.19e−16
0.3	2.92e−2	6.22e−6	1.25e−8	3.33e−16
0.6	1.54e−2	5.85e−6	9.54e−9	1.11e−16
0.9	4.73e−2	1.66e−5	1.61e−8	3.89e−16

### *Example 3*

The following nonlinear integral equation is considered

$$u(x)+\int _0^1xe^{u(y)}dy=xe^{1},$$

with $0<x<1$ and the exact solution $u(x)=x.$

In Table 3, we choose $\sigma _i=i/10, (i=0,1,\ldots ,10)$. In Table 4, we choose $\sigma _i=0,1/2,1,$ and $r_i, (i=0,1,\ldots ,10)$, respectively. $r_i, (i=0,1,\ldots ,10)$ are randomly selected. Table 3 shows the numerical results by using the present method, and Table 4 shows the choice of $\sigma _i$ has a great influence on the accuracy of numerical solutions.

**Table 3:** Absolute errors for Example 3

*x*	*n* = 4	*n* = 8	*n* = 16	*n* = 32	*n* = 64
0.2	1.21e−3	3.02e−4	7.53e−5	1.88e−5	4.70e−6
0.4	2.43e−3	6.03e−4	1.51e−4	3.76e−5	9.41e−6
0.6	3.64e−3	9.05e−4	2.26e−4	5.65e−5	1.41e−5
0.8	4.86e−3	1.21e−3	3.01e−4	7.52e−5	1.88e−5

**Table 4:** Absolute errors for Example 3

*x*	$n=8,\sigma _i=0$	$n=8,\sigma _i=1/2$	$n=8,\sigma _i=1$	$n=8,\sigma _i=r_i$
0.2	1.14e−2	1.12e−3	1.02e−2	5.88e−4
0.4	2.27e−2	2.24e−3	2.04e−2	1.18e−3
0.6	3.41e−2	3.36e−3	3.06e−2	1.76e−3
0.8	4.54e−2	4.48e−3	4.07e−2	2.35e−3

## Conclusions

Based on the idea of the integral mean value theorem and Newton iteration, a novel algorithm is constructed to solve the nonlinear Fredholm integral equations of the second kind. The convergence and the error of numerical results have been analyzed. By the obtained numerical results, we know the algorithm is feasible and valuable.
